# Association of Immune and Metabolic Receptors *C5aR* and *C5L2* with Adiposity in Women

**DOI:** 10.1155/2014/413921

**Published:** 2014-01-12

**Authors:** Pegah Poursharifi, Reza Rezvani, Abhishek Gupta, Marc Lapointe, Picard Marceau, André Tchernof, Katherine Cianflone

**Affiliations:** ^1^Centre de Recherche de l'Institut Universitaire de Cardiologie et de Pneumologie de Québec (CRIUCPQ), Laval University, Y4323, 2725 Chemin Ste-Foy, Québec, QC, Canada G1V 4G5; ^2^Department of Medicine, Laval University, 1050 Avenue de la Médecine, Québec, QC, Canada G1V 0A6

## Abstract

Adipose tissue receptors *C5aR* and *C5L2* and their heterodimerization/functionality and interaction with ligands *C5a* and acylation stimulating protein (ASP) have been evaluated in cell and rodent studies. Their contribution to obesity factors in humans remains unclear. We hypothesized that *C5a* receptors, classically required for host defense, are also associated with adiposity. Anthropometry and fasting blood parameters were measured in 136 women divided by body mass index (BMI): normal/overweight (≤30 kg/m^2^; *n* = 34), obese I (≤45 kg/m^2^; *n* = 33), obese II (≤51 kg/m^2^; *n* = 33), and obese III (≤80 kg/m^2^; *n* = 36). Subcutaneous and omental adipose tissue *C5aR* and *C5L2* expression were analysed. *C5L2* expression was comparable between subcutaneous and omental across all BMI groups. Plasma ASP and ASP/omental *C5L2* expression increased with BMI (*P* < 0.001 and *P* < 0.01, resp.). While plasma *C5a* was unchanged, *C5aR* expression decreased with increasing BMI in subcutaneous and omental tissues (*P* < 0.01 and *P* < 0.05, resp.), with subcutaneous omental depots. Omental *C5L2/C5aR* ratio increased with BMI (*P* < 0.01) with correlations between *C5L2/C5aR* and waist circumference, HDL-C, and adiponectin. Tissue and BMI differences in receptors and ligands, particularly in omental, suggest relationship to metabolic disturbances and highlight adipose-immune interactions.

## 1. Introduction

The classical heat insulator and fat storage organ, adipose tissue, is now recognized as an active metabolic regulator, which synthesizes and/or secretes various cytokines and hormones [1]. White adipose tissue is found subcutaneously throughout the body, while internal organs are surrounded by omental or visceral adipose tissue. Increased omental fat mass (central obesity) strongly contributes to the pool of circulating inflammatory adipokines associated with metabolic complications such as dyslipidemia, insulin resistance, type 2 diabetes, and increased risk of metabolic syndrome [2, 3]. The precise mechanisms linking omental depots and metabolic complications are yet unclear. However, recent emphasis on “immunometabolism” has become a major focus of both metabolic and immunologic research, with the demonstration of crosstalk between adipokines and the innate immune system (including complement components) [4, 5].

One example of a protein bridging immunity and metabolism is acylation stimulating protein (ASP), an adipose tissue-derived hormone, which is the product of complement component *C3* cleavage [6]. Circulating ASP levels are associated with atherosclerosis, type 2 diabetes, and are increased by several fold in obese versus normal weight controls [6, 7]. ASP manifests its insulin-like effects on differentiated human adipocytes via the receptor *C5L2* [8]. However, the most potent anaphylatoxin, complement *C5a*, also binds *C5L2*, as well as its own classical receptor *C5aR* [9, 10]. *C5a* is a multifunctional protein, stimulating chemotaxis, enzyme/cytokine release, and the respiratory burst [10]. Pathological conditions such as sepsis and various immunoinflammatory disorders are accompanied by increases in circulating *C5a* [11–13].

*C5L2*, initially proposed as a nonfunctional receptor, has been shown to be actively involved in inflammatory conditions such as insulin resistance, asthma, and coronary artery disease [14, 15]. Accumulating evidence demonstrates direct interactions between *C5L2* and *C5aR* [16], and this has been implicated in inflammatory conditions such as sepsis [13, 17]. Recently, the well-defined proinflammatory *C5a-C5aR* pathway has been targeted for pharmacological therapy via inhibition of *C5* cleavage, *C5a* blocking antibodies or *C5aR* antagonists for treatment of sepsis, cardiovascular diseases, autoimmune disorders, asthma, and psoriasis [18, 19]. However, the consequences of interfering with the *C5a-C5aR* pathway could also have a metabolic impact on *C5L2* signaling and this requires clear knowledge and consideration of *C5L2* and its ligand- and tissue-specific effects.

Given the documented homo- and heterodimerization of *C5L2* and *C5aR*, and the resulting potential alternative signaling pathways in adipocytes [16, 20, 21], the *C5L2/C5aR* ratio was evaluated in a human study. We hypothesized that proportional expression of *C5L2* relative to *C5aR* would vary between specific adipose tissue depots and would be influenced by obesity. As such, this was evaluated in both subcutaneous and omental adipose tissues and its association with metabolic factors in adult women over a wide range of BMI values was investigated.

## 2. Materials and Methods 

### 2.1. Subjects

Samples were obtained from (i) severely obese women who had undergone weight-loss surgery (biliopancreatic diversion, BPD) at the CRIUCPQ (Centre de Recherche de l'Institut Universitaire de Cardiologie et Pneumologie de Québec) and (ii) healthy women who had undergone elective surgery at the Gynecology Unit, Laval University Medical Center. All patients met the following eligibility criteria for entry into the study: women aged between 21 and 69 years, nondiabetic, not taking medication for dyslipidemia, had not previously undergoing ovarectomy, and with availability of matched blood and adipose tissue samples. The total group contained samples from 136 women, with body mass indexes (BMI; weight/height^2^) ranging from 19.5 to 78.9 kg/m^2^. Research protocols were approved by the CRIUCPQ and CHUL institutional review boards. Subjects with severe obesity were recruited through our institution-approved tissue bank for the study of the causes and consequences of obesity (http://www.criucpq.ulaval.ca/index.php/en/tissue-bank). All the participants provided written informed consent prior to the enrollment.

### 2.2. Study Design

Individuals were classified into quartile groups based on their BMI. In addition to the normal/overweight category (*n* = 34), defined as a BMI of less than or equal to 30 kg/m^2^, additional obese groups were defined as follows: BMI > 30 to BMI ≤ 45 (obese group I; *n* = 33), BMI > 45 to BMI ≤ 51 (obese group II; *n* = 33), and BMI > 51 to BMI ≤ 80 (obese group III; *n* = 36).

### 2.3. Physical Measures

Anthropometric measurements including body weight, height, and waist circumference were measured the day before surgery. BMI was calculated by standard formula (weight in kilograms divided by height in meters squared). Blood pressure was measured in the right arm with the participant seated after at least 5 minutes of rest. The average of two sequential measures was used.

### 2.4. Blood Lipids and Hormones

Blood samples were collected in a fasted state and immediately centrifuged to obtain plasma. Biochemical parameters (fasting plasma glucose, triglyceride, total cholesterol, low density lipoprotein cholesterol (LDL-C), high density lipoprotein cholesterol (HDL-C), apolipoprotein B (ApoB), and apolipoprotein A1 (ApoA1)) were measured by the hospital biochemistry laboratory (IUCPQ and CHUL, QC) according to validated clinical procedures. The remaining plasma was transferred to the research laboratory for the following measurements: adiponectin by commercial radioimmunological assay according to the manufacturers' protocol (Millipore, MA) and *C5a* by commercially available ELISA kit (BD Biosciences, San Jose, CA). Plasma ASP concentration was measured using an in-house sandwich ELISA following previously published methodology [22].

### 2.5. Tissues

Adipose tissue samples were obtained from the subcutaneous and omental depots during surgery. The adipose tissue samples were rinsed with sterile Krebs-Ringer-HEPES buffer, placed in liquid nitrogen, and frozen at −80°C until analysis.

### 2.6. RNA Extraction and Real-Time qPCR

Omental and subcutaneous adipose tissues (maximum 100 mg) were homogenized in Qiazol lysis reagent (Qiagen, Mississauga, ON). Following mRNA extraction using RNeasy Plus Universal Mini Kit (Qiagen, Mississauga, ON), a total amount of 0.1 *μ*g RNA was reverse transcribed to cDNA (final volume of 20 *μ*L) using QuantiTect Reverse Transcription Kit (Qiagen, Mississauga, ON). Genomic DNA contamination was eliminated by DNase treatment included in QuantiTec Reverse Transcription Kit. All Real-Time PCR reactions were performed in a 25 *μ*L mixture containing cDNA (1 *μ*L), RT^2^ SYBR Green qPCR Master Mix (Qiagen, Mississauga, ON) (12.5 *μ*L), RNase-free water (10.5 *μ*L), and 0.5 *μ*L of each primer. Negative controls (without cDNA or reverse transcription) were also performed. Three-step PCR amplification was conducted using CFX96 Real-Time PCR Detection System (Bio-Rad Laboratories, Mississauga, ON) with the following instrumental settings: a denaturation step at 95°C for 10 min, 39 cycles of 95°C for 15 s, 55°C for 40 s, 72°C for 30 s, and a final extension step of 95°C for 10 s. *C5L2* primers were purchased from Qiagen (GPR77: QT00243971, QuantiTect Primer Assay, Qiagen, Mississauga, ON). *C5aR* and GAPDH primers were ordered from Alpha-DNA (Montreal, QC) with the following sequences: *C5R1* forward: 5′-GCCCAGGAGACCAGAACAT-3′ reverse: 5′-TATCCACAGGGGTGTTGAGG-3′, GAPDH forward: 5′-AAGGTGAAGGTCGGAGTCAA-3′ reverse: 5′-AATGAAGGGGTCATTGATGG-3′. Results were analysed by the ΔΔCt relative quantification method using Bio-Rad CFX manager software (version 1.5) (Bio-Rad Laboratories, Mississauga, ON) and normalized to *GAPDH *(housekeeping gene). All procedures followed Minimum Information for Publication of Quantitative Real-Time PCR Experiments (MIQE) guidelines including specificity, appropriate controls, and assay performance [23].

### 2.7. Statistical Analysis

All anthropometric measurements, plasma parameters, and adipose tissue gene expression data are expressed as mean ± SEM for normally distributed data and median interquartile range for nonnormally distributed data. Groups were compared by two-way analysis of variance (ANOVA) followed by Bonferroni posttest, one-way ANOVA, or Student's *t*-test, as indicated, using GraphPad Prism 5 (GraphPad Software, Inc., San Diego, CA). Relationships between variables in each group were assessed by linear regression analysis using Pearson correlation. Statistical significance was indicated as follows: *P < 0.05, **P < 0.01, and ***P < 0.001, where *P* NS indicates no significant difference.

## 3. Results

### 3.1. Anthropometric and Blood Characteristics of Normal/Overweight and Obese Groups

Table 1 shows the anthropometric, lipid, and hormone characteristics of the normal/overweight and obese groups I, II, and III. There was no significant difference in the average age of the women in these four groups.In addition to the expected differences in BMI (assigned groups), there were marked differences in waist circumference and systolic and diastolic blood pressure between normal/overweight group and obese groups (*P* < 0.001). While there was no significant difference between normal/overweight and obese groups (I, II, and III) for fasting glucose, total cholesterol, LDL-C, and ApoB, the obese groups had, however, significantly lower HDL-C, ApoA1, and adiponectin than normal/overweight women.

### 3.2. Circulating ASP and ASP to *C5L2* Ratio Are Associated with Adiposity

**Figure 1:**
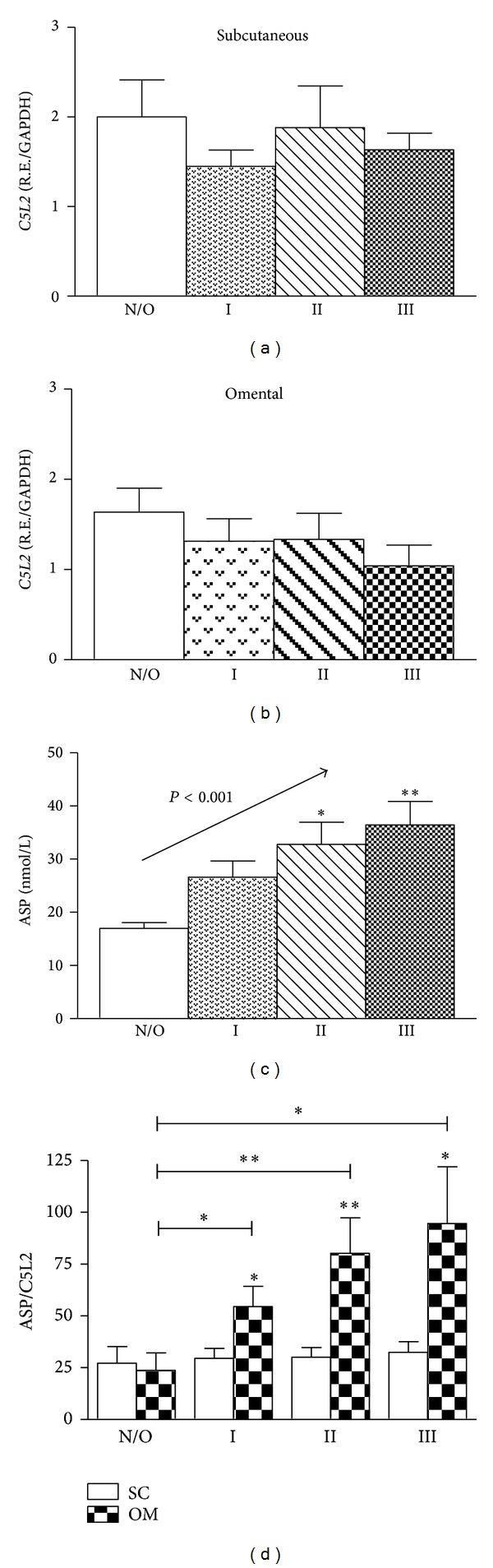
Circulating ASP and ASP to *C5L2* ratio are associated with adiposity. Subcutaneous (a) and omental (b) *C5L2* mRNA expression for normal/overweight (open bar), obese group I (dotted bar), obese group II (striped bar), and obese group III (checkered bars), (c) plasma ASP for normal/overweight (open bar), obese group I (dotted bar), obese group II (striped bar), and obese group III (checkered bars), and (d) ASP/*C5L2* ratio in subcutaneous adipose tissue (open bars) and omental adipose tissue (checkered bars). Results are expressed as means ± SEM; *n* = 33–36 per group. Statistical differences were determined by Student's *t*-test and one-way ANOVA, for normal/overweight versus obese groups and for SC versus OM groups, where *P < 0.05 and **P < 0.001.

As shown in Figures 1(a) and 1(b), no significant differences in *C5L2* expression were observed between normal/overweight and obese groups in either subcutaneous or omental adipose tissues, and there was no significant difference between subcutaneous versus omental (*P* = NS, 2 way ANOVA). However, relative to body mass index, plasma ASP increased by up to twofold in the obese III group (Figure 1(c), linear trend *P* < 0.001). The ASP/*C5L2* ratio, representing the ligand/receptor ratio, was calculated individually for each subject, and, as shown in Figure 1(d), this ratio increased proportionately to BMI in omental adipose tissue (up to 300% in group III versus normal/overweight, linear trend *P* < 0.01). By contrast, in subcutaneous adipose tissue, the ASP/*C5L2* ratio remained comparable in all four groups with no significant differences between normal/overweight and obese groups (Figure 1(d)).

### 3.3. *C5aR* Expression in Both Subcutaneous and Omental Adipose Tissue Is Downregulated in Obesity

**Figure 2:**
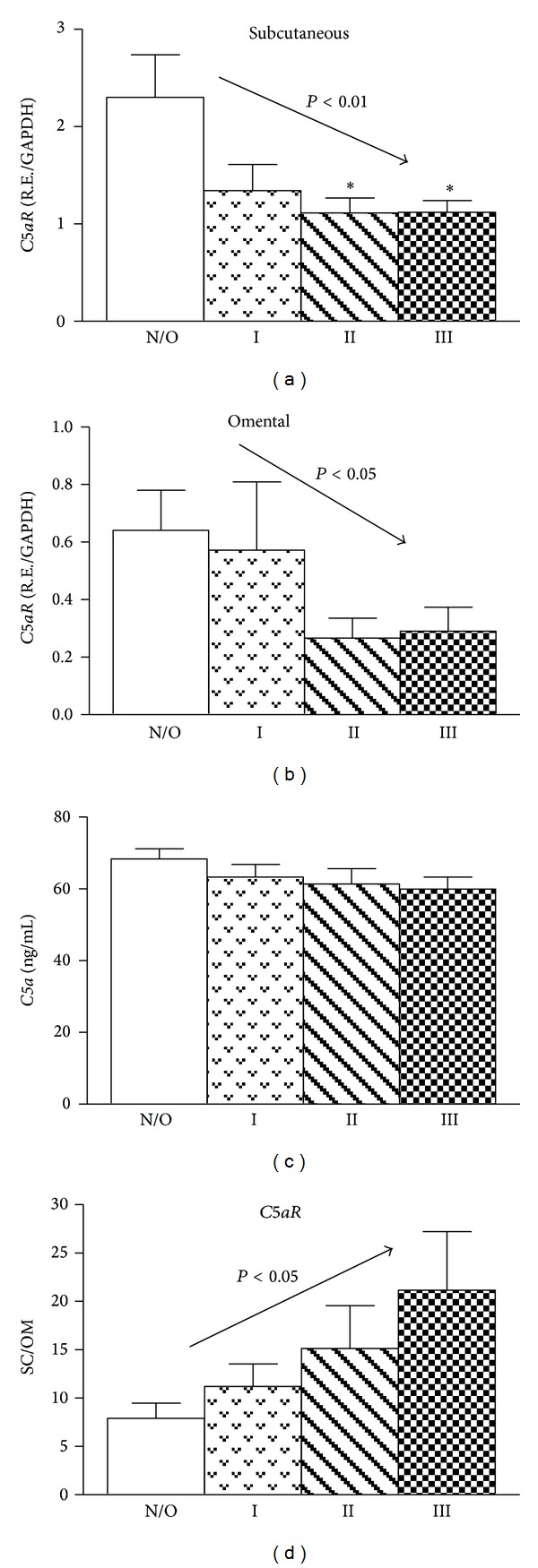
*C5aR* expression in both subcutaneous and omental adipose tissues is downregulated in obesity. Subcutaneous (a) and omental (b) *C5aR* mRNA expression for normal/overweight (open bar), obese group I (dotted bar), obese group II (striped bar), and obese group III (checkered bars), (c) plasma *C5a* for normal/overweight (open bar), obese group I (dotted bar), obese group II (striped bar), and obese group III (checkered bars), and (d) subcutaneous/omental *C5aR* expression for normal/overweight (open bar), obese group I (dotted bar), obese group II (striped bar), and obese group III (checkered bars). Results are expressed as means ± SEM; *n* = 33–36 per group. Statistical differences were determined by Student's *t*-test and one-way ANOVA, for normal/overweight versus obese groups, where *P < 0.05.

As shown in Figure 2, *C5aR* expression decreased with increasing BMI in both subcutaneous and omental adipose tissues (Figures 2(a) and 2(b), linear trend *P* < 0.01 and *P* < 0.05, resp.). In contrast to ASP, there was no significant difference in *C5a* concentration between normal/overweight and all levels of obesity (Figure 2(c)). Interestingly, *C5aR* expression in subcutaneous tissue was significantly greater than omental tissue at all levels of obesity (*P* < 0.05, 2 way ANOVA). Further, as demonstrated in Figure 2(d), although *C5aR* expression decreased in both tissues with increasing obesity levels, there was a proportionally greater decrease in omental tissue, such that the subcutaneous/omental ratio of *C5aR* expression tended to increase with increasing levels of obesity (linear trend *P* < 0.05).

### 3.4. Omental *C5L2/C5aR* Ratio as a Potential Marker of Obesity

**Figure 3:**
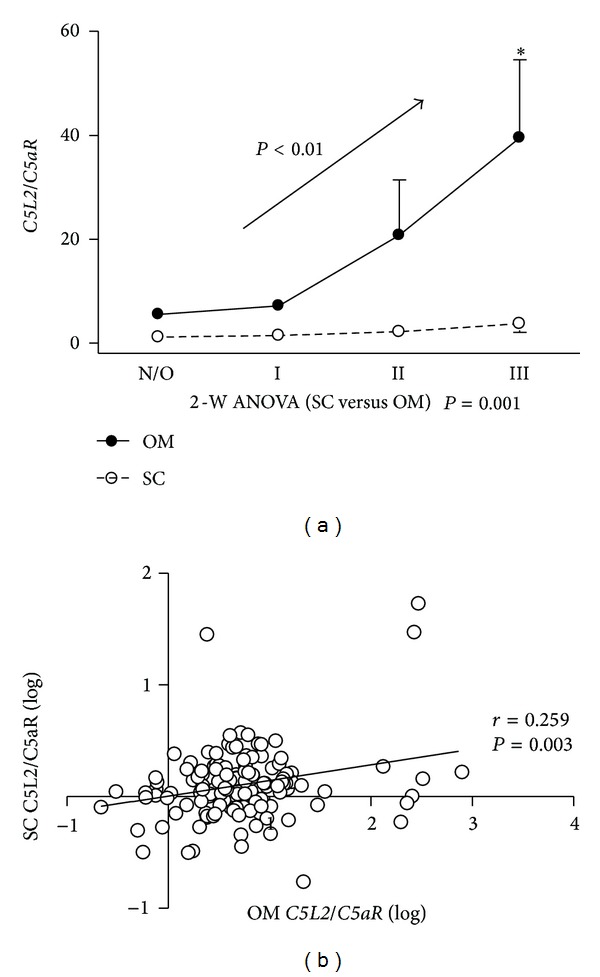
Omental *C5L2/C5aR* ratio in relation to obesity.(a)* C5L2/C5aR* ratio in omental adipose tissue (solid circles) versus subcutaneous adipose tissue (open circles), (b) linear regression analysis of subcutaneous versus omental *C5L2/C5aR* ratio (log-log values). Reported *r* and *P* values were calculated by Pearson correlation. Data are expressed as mean ± SEM and were compared by Student's *t*-test and one-way ANOVA (versus normal/overweight group), as well as two-way ANOVA (subcutaneous versus omental) where *P < 0.05.

As *C5L2* and *C5aR* heterodimerize [16], the ratio of *C5L2/C5aR* was evaluated. As shown in Figure 3(a), omental *C5L2/C5aR* ratio differed significantly from subcutaneous *C5L2/C5aR* ratio (*P* = 0.0012, 2 way ANOVA). While the *C5L2/C5aR* ratio remained constant in subcutaneous tissue over the range in BMI, in omental tissue there was a significant increase in *C5L2/C5aR* ratio (linear trend *P* < 0.01) (Figure 3(a)). There was also a significant increase in ASP/*C5a* ratio with increasing obesity (linear trend *P* < 0.05; data not shown). Additionally, a positive correlation (*r* = 0.259, *P* = 0.003) between *C5L2/C5aR* ratios was observed between subcutaneous and omental adipose tissue (Figure 3(b)).

### 3.5. *C5L2/C5aR* Is Associated with Anthropometric Indices, HDL, and Adiponectin

**Figure 4:**
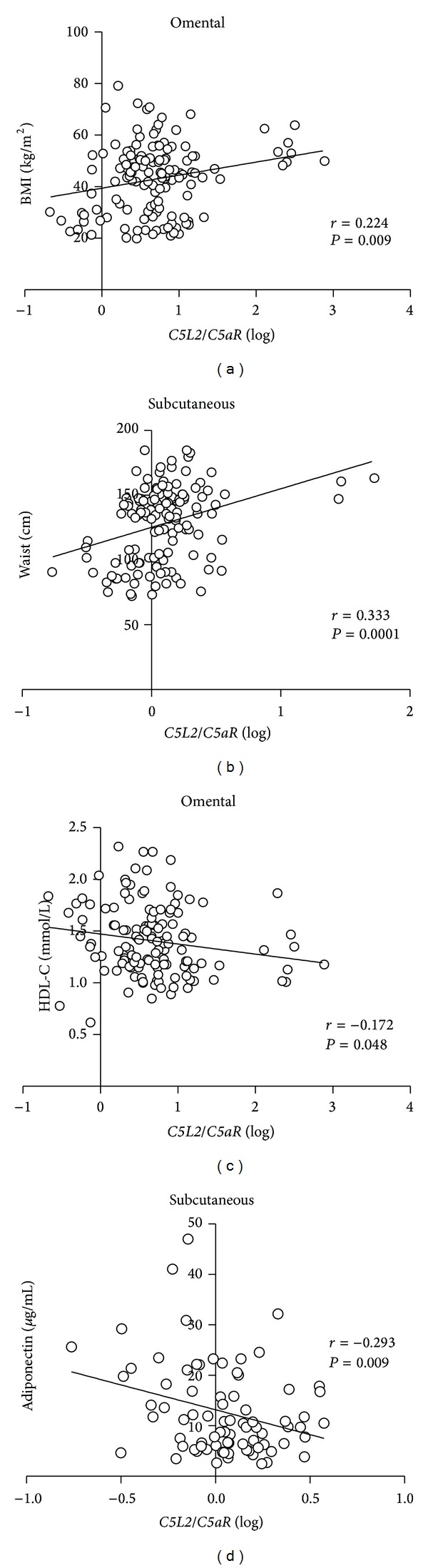
*C5L2/C5aR* ratio is associated with anthropometric indices, HDL, and adiponectin. Linear regression analysis of omental *C5L2/C5aR* versus BMI (a), subcutaneous *C5L2/C5aR* versus waist circumference (b), omental *C5L2/C5aR* versus HDL-C (c), and subcutaneous *C5L2/C5aR* versus adiponectin (d). Reported *r* and *P* values were calculated by Pearson correlation.

Omental* C5L2/C5aR* demonstrated positive associations with anthropometric parameters such as weight (*r* = 0.262, *P* = 0.002), BMI (*r* = 0.223, *P* = 0.009, Figure 4(a)), and waist circumference (*r* = 0.228, *P* = 0.009). Comparable significant correlations were also found between subcutaneous *C5L2/C5aR* ratio and weight (*r* = 0.318, *P* = 0.0001), BMI (*r* = 0.300, *P* = 0.0004), and waist circumference (*r* = 0.333, *P* = 0.0001; Figure 4(b)). Furthermore, there were significant inverse relationships between omental *C5L2/C5aR* and plasma HDL-C (*r* = −0.172, *P* = 0.048; Figure 4(c)), as well as subcutaneous *C5L2/C5aR* ratio and circulating adiponectin (*r* = −0.293, *P* = 0.009; Figure 4(d)).

## 4. Discussion

Despite the marked increase in bariatric surgery procedures in the last decade, the rate of severe obesity continues to increase and exceeds that of moderate obesity in the United States [24]. To add to that, recent clinical evidence indicates that complications stemming from obesity are not only related to the extent of fat accumulation but also to the pattern of fat distribution [25, 26]. More recently, research detailing the contribution of the immune system to the observed obesity-induced inflammation has enhanced our understanding of this multifactorial disorder. This current study adds to this, with the emerging concept that *C5a* and its receptor *C5aR*, traditionally considered to be required only for host defense, are also associated with adipose tissue metabolic dysfunction, as discussed below. However, the limitations of the study should be noted: all data were obtained in women, and due to the cross-sectional nature of the analyses, cause-and-effect relationships cannot be determined. In addition, based on the limited availability of frozen adipose tissue in small quantities, the present study relied on *C5aR* and *C5L2* mRNA expression without addressing the possible posttranslational modifications and protein levels of the examined receptors. Further, as only frozen tissue was available, direct ligand functional assays in tissues could not be performed.

It has been repeatedly shown that circulating ASP levels are altered in response to pathophysiological conditions in humans, including augmentation in obesity, cardiovascular disease, and type II diabetes (even in the absence of obesity), and their reduction with exercise or weight loss [6, 7]. In the present study, not unexpectedly, plasma ASP increased markedly with increasing BMI. While the associated consequences of this increase in ASP in humans remain speculative, its causes can at least be partially explained by the observations that dietary fatty acids, chylomicrons, and insulin can increase ASP production [27, 28]. Furthermore, obesity-associated adipose tissue metabolic complications such as an imbalance in lipogenesis/lipolysis, delayed triglyceride clearance, dysregulated adipokine, and *C3* (ASP precursor) production, as well as fat accumulation, all exert profound impacts on ASP secretion [6, 27]. Based on *in vitro* and *in vivo* experiments, ASP stimulates triglyceride synthesis and fat storage in adipose tissue, while disruption of the ASP-*C5L2* pathway in mice resulted in delayed lipid clearance and redistribution of lipids towards skeletal muscle for oxidation, a consequence which has been shown to be reversible with ASP administration [6, 29].

Beyond the proposed role of *C5L2* in lipid storage and adipose metabolism, Huber-Lang et al. demonstrated a reduction of C5L2 protein content in neutrophils during sepsis [30]. Likewise, *C5L2 *expression was downregulated in neutrophils from patients with Familial Mediterranean Fever (an autoinflammatory syndrome) [31]. Furthermore, Raby et al. proposed a negative regulatory effect of TLR on *C5L2* expression following *C5a* stimulation [32]. Alteration of *C5L2* expression in inflammatory-based disorders together with previous evidence that *C5L2* expression in adipocytes is regulated by differentiation, TNF-*α*, and rosiglitazone all indicates a potent pathophysiological role for the ASP-*C5L2* pathway in adipose tissue inflammation [33, 34]. Interestingly, Fisette et al. demonstrated that a combined high fat-high sucrose diabetogenic diet worsens the inflammatory state of *C5L2 *(−/−) mice [35]. This phenotype, demonstrated experimentally in mice, is consistent with metabolic features of the obese women in the current study, exhibiting increased plasma ASP and a corresponding ASP/*C5L2* ratio increment in the omental fat depot. This coupling of increased ligand to decreased receptor is suggestive of a downregulated pathway, which could be consistent with an “ASP resistant” state in humans. In addition, as ASP is an important regulator of postprandial lipemia [6] an increase in basal plasma lipids in obesity could be the consequence of putative ASP resistance, analogous to the hyperglycemia in insulin-resistant states. A recent publication has provided a “proof-of-concept” of ASP resistance in diet-induced obesity [36]. Feeding wild-type mice a high fat-high sucrose diet led to a decrease in *C5L2* expression, increased plasma ASP, and reduced ASP functional activity as evidenced by decreased *in vivo* ASP-mediated postprandial fat clearance and decreased *in vitro* ASP-mediated Akt phosphorylation in gonadal fat depots following ASP injection [36]. Taken together with ASP proinflammatory effects on adipose tissue, such as stimulation of inflammatory cytokine production and macrophage infiltration/M1 polarization [37, 38], the altered ASP/*C5L2* ratio in omental tissue may be both reflective of impaired ASP functionality as well as contributing to the dyslipidemia and metabolic disturbances of obesity.

Recent studies have demonstrated that *C5aR *(−/−) mice have decreased adipose tissue weight, lower plasma lipids, and reduced fat storage regardless of diet [39]. Further, administration of *C5aR*-selective antagonists in diet-induced obese rats resulted in weight loss and improvement in insulin resistance and adipose tissue inflammation [40]. Other studies indicate that *C5a* stimulates increased fatty acid [20, 40] and glucose uptake in adipocytes, while inhibiting cAMP stimulation and lipid lipolysis [40]. These findings highlight the recently identified role of *C5a-C5aR* in metabolic disorders such as obesity, while supporting an anti-inflammatory role for *C5aR* antagonists in animal models of inflammatory diseases [41, 42]. Blogowski et al. demonstrated a constant plasma level of *C5a* between lean, overweight, and obese individuals [43], consistent with data presented here. However, the marked downregulation of *C5aR* in both subcutaneous and omental depots of obese women in this study raises an interesting question: could *C5aR* antagonists, which are currently being used in Phase I and II clinical trials for treatment of asthma, psoriasis, and rheumatoid arthritis [19], have additional metabolic-related effects? Further, the potential *C5L2* regulatory impact on *C5aR* also needs to be taken into account.

We had hypothesized a potential physiological role for the *C5L2*/*C5aR* ratio, based on the following evidence: (i) *in vitro* studies on transfected cells have indicated the presence of constitutive *C5aR-C5L2* heterodimers, in addition to evidence of cell-specific localization and the cointernalization/colocalization of *C5aR* and *C5L2* following *C5a* or ASP treatment [16, 21, 44]; (ii) synergic contributions of both *C5aR* and *C5L2* are required for the production of G-CSF during acute inflammation [17] and the harmful consequences and lethality observed during sepsis [45]; (iii) recent publications on *C5aR* or *C5L2* knockout mice have emphasized that disruption of either receptor resulted in decreased expression of the complementary receptor in retroperirenal and gonadal adipose tissues but not in liver [39, 46]. Moreover, elevated *C5L2* expression was shown to be accompanied by a likewise increase in *C5aR* gene expression in adipose tissue, muscle, and liver of wild-type mice on a diet-induced obesity (DIO) regimen [39]. Thus, while there appears to be a coupled relationship between *C5L2* and *C5aR*, this relationship appears to be tissue-specific and can be regulated differentially based on disease conditions [31, 47]. For example, in neutrophils from patients with Familial Mediterranean Fever, *C5L2* is decreased but not* C5aR *[31]. In kidney biopsies from patients with antineutrophil cytoplasmic antibody- (ANCA-) associated glomerulonephritis, *C5aR* is downregulated but *C5L2* is upregulated [47]. In the current study, there are adipose tissue depot-specific changes leading to increases in the *C5L2/C5aR *ratio, which are also associated with obesity indicators.

This issue of receptor dimerization has been suggested to regulate many aspects of receptor function including synthesis, ligand binding, and intracellular trafficking and downstream signaling [48]. However, the heterodimerization of *C5L2-C5aR* is a recently observed phenomenon and the functional consequences with respect to signaling pathways, particularly in obesity pathophysiology, are still unexplored. In adipocytes from *C5a*RKO mice, *C5a* induces a greater increase in ERK phosphorylation than in wild-type adipocytes [20]. In the present study the strong downregulation of *C5aR* along with ASP resistance may potentially increase *C5a-C5L2* interaction which may further induce ERK phosphorylation. Interestingly, ERK/MAPK activation has been associated with the regulation of adipocyte differentiation, adiposity, high-fat diet induced obesity, and type 2 diabetes [49, 50].

It is striking that the increased ASP/*C5L2* and *C5L2/C5aR* ratios occur specifically in omental adipose tissue. We speculate that the decreased *C5aR* relative to *C5L2*, in the face of maintained *C5a* levels, could divert *C5a* towards *C5L2* interaction (promoting a proinflammatory response), simultaneously interfering with ASP action, increasing internalization/downregulation of *C5L2*, leading to compensatory increases in circulating ASP (as demonstrated in this study and others in obese subjects). *In vitro* studies in adipocytes demonstrated that the simultaneous treatment of *C5a*RKO adipocytes with ASP and *C5a* blocks the ASP-*C5L2* pathway [20]. Accordingly, we hypothesize that this interference may consequently induce a state of “ASP resistance” in omental adipocytes from obese subjects, as evidenced by increased plasma ASP concentrations. Of note, ASP binding affinity and ASP functional stimulation are more pronounced in subcutaneous versus omental adipocytes [51], which supports the potential for ASP resistance in the omental depot. This could decrease the capacity of adipose tissue to efficiently uptake postprandial glucose and free fatty acid, which, coupled with inefficient anti-inflammatory *C5L2* effects, could contribute to systemic inflammation associated with obesity and insulin resistance and is consistent with the associations with high levels of circulating lipids and lower adiponectin and HDL-C. Thus altogether, increased ASP/*C5L2* and *C5L2/C5aR* ratios in omental adipose tissue are commensurate with the known pathophysiology of omental adiposity and its role in obesity-induced metabolic alterations [26].

## 5. Conclusion

Collectively, these findings shed light on the complexity of *C5L2-C5aR* interaction, providing further insight into the immunopathology of obesity while suggesting a potential role for the *C5L2/C5aR* ratio in omental adiposity. The balance between *C5aR* and *C5L2* expression can thus be observed in the ratio of *C5L2/C5aR* and is postulated to contribute to tissue-dependent ASP resistance and the adverse physiological effects that stem from it.

## Figures and Tables

**Table 1:** Anthropometric and plasma variables of normal/overweight and obese groups.

Characteristic *N* = 136	Normal/overweight (*n* = 34)	Obese I (*n* = 33)	Obese II (*n* = 33)	Obese III (*n* = 36)
Age (yrs)	46.7 ± 0.7	42.5 ± 1.6	38.8 ± 1.2	41.4 ± 1.9
BMI (kg/m^2^)	25.1 (22.5–27.7)	41.5 (35.5–43.2)***	48.6 (46.4–49.9)***	56.4 (53.4–63.2)***
Waist circumference (cm)	87.2 ± 1.4	126.1 ± 2.7***	141.5 ± 1.5***	156.8 ± 2.7***
BP systolic (mmHg)	115 (102–126)	132 (120–140)***	138 (129–146)***	139.5 (129–145)***
BP diastolic (mmHg)	70 (60–80)	83 (73–90)***	84 (77–93)***	84 (78–93)***
Glucose (mmol/L)	5.7 (5.1–6.1)	5.4 (4.6–6.0)	5.3 (4.8–5.7)	5.7 (5.0–6.8)
Total cholesterol (mmol/L)	4.9 ± 0.1	4.8 ± 0.2	4.9 ± 0.2	4.9 ± 0.1
HDL-C (mmol/L)	1.7 (1.6–2.1)	1.5 (1.3–2.3)*	1.4 (1.2–2.2)**	1.4 (1.3–2.2)*
LDL-C (mmol/L)	2.8 ± 0.1	2.8 ± 0.2	2.8 ± 0.1	2.8 ± 0.1
Triglyceride (mmol/L)	1.5 (1.0–2.5)	1.9 (1.4–3.7)	2.2 (1.5–4.4)*	2.0 (1.4–3.8)
ApoB (g/L)	1.1 (0.9–1.4)	1.1 (0.7–2.2)	1.1 (0.7–1.6)	1.1 (0.7–1.9)
ApoA1 (g/L)	1.6 (1.4–1.8)	1.4 (1.2–1.8)**	0.9 (0.7–1.9)***	1.0 (0.9–1.9)***
Adiponectin (*μ*g/mL)	22.6 (19.1–47.0)	16.8 (7.1–32.2)**	10.5 (6.6–12.2)***	8.5 (5.4–10.5)***

136 patients were classified into four weight categories based on their BMI: normal/overweight (BMI ≤ 30; *n* = 34), obese group I (BMI > 30 to BMI ≤ 45; *n* = 33), obese group II (BMI > 45 to BMI ≤ 51; *n* = 33), and obese group III (BMI > 51 to BMI ≤ 80; *n* = 36). Values are expressed as mean ± SEM for normally distributed data and median interquartile range for nonnormally distributed data. Results were compared by one-way ANOVA versus normal/overweight group where *P < 0.05, **P < 0.01, and ***P < 0.001. BMI: body mass index; BP: blood pressure; HDL-C: high density lipoprotein cholesterol; LDL-C: low density lipoprotein cholesterol; apoB: apolipoprotein B; apoA1: apolipoprotein A1.

## Abbreviations

ASP: Acylation stimulating protein
*C5L2*: *C5aR*-like receptor 2
GPCR: G protein-coupled receptor.
